# Fostering population-based cohort data discovery: The Maelstrom Research cataloguing toolkit

**DOI:** 10.1371/journal.pone.0200926

**Published:** 2018-07-24

**Authors:** Julie Bergeron, Dany Doiron, Yannick Marcon, Vincent Ferretti, Isabel Fortier

**Affiliations:** 1 Research Institute of the McGill University Health Centre, Montreal, Quebec, Canada; 2 Swiss Tropical and Public Health Institute, Basel, Switzerland; 3 University of Basel, Basel, Switzerland; 4 Research Center of the Sainte-Justine University Hospital, Montreal, Quebec, Canada; Karolinska Institutet, SWEDEN

## Abstract

**Background:**

The lack of accessible and structured documentation creates major barriers for investigators interested in understanding, properly interpreting and analyzing cohort data and biological samples. Providing the scientific community with open information is essential to optimize usage of these resources. A cataloguing toolkit is proposed by Maelstrom Research to answer these needs and support the creation of comprehensive and user-friendly study- and network-specific web-based metadata catalogues.

**Methods:**

Development of the Maelstrom Research cataloguing toolkit was initiated in 2004. It was supported by the exploration of existing catalogues and standards, and guided by input from partner initiatives having used or pilot tested incremental versions of the toolkit.

**Results:**

The cataloguing toolkit is built upon two main components: a metadata model and a suite of open-source software applications. The model sets out specific fields to describe study profiles; characteristics of the subpopulations of participants; timing and design of data collection events; and datasets/variables collected at each data collection event. It also includes the possibility to annotate variables with different classification schemes. When combined, the model and software support implementation of study and variable catalogues and provide a powerful search engine to facilitate data discovery.

**Conclusions:**

The Maelstrom Research cataloguing toolkit already serves several national and international initiatives and the suite of software is available to new initiatives through the Maelstrom Research website. With the support of new and existing partners, we hope to ensure regular improvements of the toolkit.

## Introduction

In the last decades, millions of citizens across the world contributed time, information and biological specimens to population-based cohorts, which in turn led to major scientific progress and to a better understanding of the relation between numerous risk factors and health outcomes. However, many cohort databases remain under-exploited. To address this issue and speed up discovery, it is essential to offer timely access to cohort data and samples[1–3]. Providing the scientific community with open information about existing research data is an important step toward optimizing usage of these unique scientific resources. However, even for well-known cohorts, specific information on samples and data collected is often either not publicly available or in a format that does not allow to easily understand study design and content. The lack of accessible and structured documentation thus creates major barriers for investigators interested in understanding, properly interpreting and analyzing longitudinal study data[1].

A number of study-specific or network catalogues have been developed over the years to promote discoverability of data and samples. The majority have been developed to answer the data documentation needs of individual studies[4–6]. However, the number of research networks co-analyzing data *across* studies has considerably increased in the past decade, leading to the implementation of central portals to document groups of studies[7–9]. The objectives of these catalogues vary, and they differ in the level of details they provide on the studies, variables and samples collected. But to truly unleash innovative research agendas and leverage usage of existing data, such catalogues need to be comprehensive and user friendly enough to easily estimate whether data: (1) is accessible to external researchers, (2) might serve to answer specific research questions (e.g. level of physical activity measured with a specific scale), and, when relevant, (3) is similar enough to enable co-analysis across multiple studies. Such criteria have been promoted in scientific data management and stewardship guidelines such as the recently published FAIR principles[3]. An additional feature particularly useful is the access to summary statistics on study subjects, such as the number of participants presenting specific characteristics (e.g. diseases or exposures).

Open source and commercial software have been developed to support the creation of data and metadata portals[10–14]. Such software offers solutions for describing datasets and finding relevant data through searching and browsing features. However, most software applications are not specifically designed for answering the practical requirements of cohorts and networks of cohorts. Therefore, individual research initiatives often need to adapt existing software or develop in-house solutions[15,16]. But developing a metadata portal is resource intensive and as generic solutions are rarely used, interoperability across partner initiatives is limited. If we are to foster a more open approach to research and optimize data discovery, we should provide access to interoperable, flexible and cost-effective software solutions to support cataloguing of longitudinal cohort data.

The present paper describes the approach and software developed by the Maelstrom Research team to answer the need for a general and customizable solution to support the creation of comprehensive and user-friendly study- and network-specific catalogues used to leverage epidemiological research making use of cohort data.

## Methods

Development of the Maelstrom Research cataloguing toolkit was initiated in 2004[17]. It was guided by the exploration of existing catalogues and the feedback gathered at workshops addressing the needs of partner initiatives and working sessions evaluating incremental versions of the toolkit pilot-tested by our partners.

### Exploring existing resources

Informal literature and Internet searches supplemented by references from key informants allowed identifying existing epidemiological study catalogues. The searches were undertaken in Ovid (Embase, Health and Psychosocial Instruments, Ovid Healthstar, Ovid MEDLINE(R) Versions, PsycINFO, Social Work Abstracts, NASW Clinical Register), PubMed, Web of Science, Scopus, ScienceDirect databases and Google search engine using a range of keywords including “metadata registry, metadata catalogue, metadata repository, metadata standard, metadata model, health databases, cohort, population-based studies, software”. Properties of all relevant catalogues were explored, with a focus on cohort-specific metadata repositories. As such, the search targeted catalogues which included cohorts or longitudinal population-based studies; documented multiple cohorts; included at least a minimal description of the cohort designs; and were accessible online (with or without protected access). Catalogues already making use of the Maelstrom Research toolkit were excluded.

A total of 126 catalogues were identified, 20 of which corresponded to the profile described above. Some of the catalogues identified in the search were excluded because they did not document epidemiological cohorts (e.g. eleMap[18] is a catalogue of phenotypes, DataOne[19] is a catalogue of environmental data), only documented individual cohorts as opposed to groups of cohorts (e.g. MIDUS[20,21]), or already used the Maelstrom Research toolkit (e.g. BioSHaRE[22,23], IALSA[24], MINDMAP[25,26]). Exploration of the catalogues’ content was achieved by a research assistant to document the framework used to describe the cohort profile, specific fields used to document information, software applications used, and study and variable search models. The information was then validated by the coordinator responsible for catalogue development. Information was retrieved by accessing and browsing the online catalogues and when relevant, discussing with individuals managing these catalogues.

### Developing and piloting the toolkit

Development of the Maelstrom Research cataloguing toolkit was achieved in collaboration with investigators and researchers making use of cohort data, as well as with international experts with various backgrounds (e.g. epidemiologists, computer scientists, statisticians, ethicists, data librarians). Using an iterative review and consensus approach, a subgroup of epidemiologists and computer scientists established guiding principles to develop maturing versions of the toolkit. The following prerequisite guided development: the toolkit had to serve the needs of both, individual studies and study networks. For individual cohorts, a cost-effective solution to disseminate information and leverage use of available data was sought. For study networks, the toolkit had to allow assessing the compatibility of data across studies and documenting harmonized datasets. It needed to also include a complementary variable classification index to facilitate variable search. In addition, the metadata model was required to be compatible with existing standards (e.g. Data Documentation Initiative (DDI)[27]), whenever possible. It was also deemed essential to provide a simple and flexible tool allowing the documentation of studies and variables dictionaries with varying levels of completeness and diverse data formats (e.g. SAS, SPSS, STATA). Finally, the toolkit needed to be accessible to all and thus, offer free, open-source and customizable software applications. To ensure short-term applicability, development was guided by the specific needs of Maelstrom Research’s partner projects. Since 2004, maturing versions of the toolkit were produced and tested by these projects (Table 1). Throughout, comments and suggestions from investigators of these initiatives were integrated in a central repository. At least once a year, the most pressing or crucial demands for improvements were selected and the toolkit was, and still is customized to answer these requests. Improved versions of the toolkit are therefore regularly generated and tested by users.

**Table 1:** Initiatives having used or piloted iterative versions of the Maelstrom Research cataloguing toolkit (2004–2017).

Study or study network [ref]	Number of studies	Country	Start year	Research focus
P^3^G[17]	164	International	2004	Various diseases
CLSA[28,29]	1	Canada	2009	Healthy aging
IMPCC[30]	13	Spain	2010	Cancer
BioSHaRE [22,23]	15	Europe	2011	Various diseases
IALSA[24]	111	International	2012	Aging
PHQE[31]	24	International	2012	Various diseases
BBMRI-LPC[32]	20	Europe	2013	Various diseases
CPTP[33,34]	5	Canada	2013	Various diseases
InterConnect[35]	209	Europe	2014	Diabetes
SPIRIT[36]	4	International	2014	Child health and development
The PREMMIUM		Canada	2014	Various diseases
ATHLOS[37]	19	International	2015	Aging
CHPT[38]	13	International	2015	Various diseases
NCI Cohort Consortium[39,40]	59	International	2015	Cancer
CONSTANCES[41,42]	1	France	2016	Various diseases
MINDMAP[25,26]	10	International	2016	Aging
NeuroDevNet[43,44]	10	Canada	2016	Neurodevelopmental diseases
ReACH[45]	26	International	2016	Developmental origins of health and disease
COHORTS.SE[46]	34	Sweden	2017	Various diseases

P^3^G, Public Population Project in Genomics and Society; CLSA, Canadian Longitudinal Study on Aging; IMPCC, Institut de Medicina Predictiva i Personalitzada del Cancer; BioSHaRE.EU, Biobank Standardization and Harmonization for Research Excellence in the European Union; IALSA, Integrative Analysis of Longitudinal Studies of Aging; PHQE, Québec-Europe Harmonization Platform for Research; BBMRI-LPC, Biobanking and Biomolecular Resources Research Infrastructure—Large Prospective Cohorts; CPTP, Canadian Partnership for Tomorrow Project; InterConnect, InterConnect: global data for diabetes and obesity research; SPIRIT, Sino-Quebec Perinatal Initiative in Research and Information Technology; The PREMMIUM, Integrated Research Platform on Mental Health and Sexually Transmitted Infections of the Université de Montréal; ATHLOS, Ageing Trajectories of Health: Longitudinal Opportunities and Synergies; CHPT, Cross-cohort Harmonization Project for Tomorrow; NCI Cohort Consortium, National Cancer Institute Cohort Consortium; CONSTANCES, Cohorte des consultants des Centres d'examens de santé; MINDMAP, Promoting mental well-being and healthy ageing in cities; NeuroDevNet, Kids brain health network; ReACH, Research Advancement through Cohort Cataloguing and Harmonization; COHORTS.SE, Swedish Cohort Consortium.

## Results

Table 2 shows existing cohort-specific catalogues identified by key informants or through Internet searches. These 20 catalogues include study descriptions, but the scope, conceptual model and completeness of the metadata fields used vary extensively. Seven (35%) of the catalogues provide a list of variables collected by studies and 2 (10%) serve as portals to access individual participants data. Only 3 (15%) annotate variables with classification schemes to facilitate the search. The potential to search information through text mining or study and variable properties depends on the structure of the metadata fields and is often limited in scope. Online or downloadable outputs (e.g. Excel tables, PDF documents) also vary, but they include: lists of studies with related properties, visualization tools outlining study characteristics (e.g. maps, tables including number of participants); list of variables and related properties; descriptive statistics (means, distribution) from participant data; and tables allowing to explore harmonization potential across studies.

**Table 2:** Characteristics of the cohort catalogues surveyed.

Initiative [ref]	Design	Country	Online access to variables	Potential to search by
Biological and BioMolecular resources Research Infrastructure (BBMRI)[47]	Various databases	Europe	None	Study properties Text in study description
Biomarker for Cardiovascular Risk Assessment in Europe (BiomarCaRE)[48]	Cohorts and clinical trials	International	None	-
Birthcohorts.net[49]	Cohorts	International	None	Study properties Text in study description Categories of information collected
B.R.I.D.G.E. TO DATA[50]	Various study designs	International	None	Study properties Text in study description Categories of information collected
Cancer Epidemiology Descriptive Cohort Database (CEDCD)[51]	Cohorts	International	None	Study properties Categories of information collected
Cohort and Longitudinal Studies Enhancement Resources (CLOSER)[52]	Cohorts	United Kingdom	Metadata only	Study properties Text in variable label Categories of information collected
Centre for Longitudinal Studies (CLS)[53]	Cohorts	United Kingdom	Metadata only	Text in variable label Categories of information collected
The Global Alzheimer’s Association Interactive Network (GAAIN)[54]	Cohorts	International	Metadata only	Study properties Text in variable label Categories of information collected
The Gateway to Global Aging Data[55]	Cohorts	International	Metadata only	Study properties Text in variable label Categories of information collected
Inter-university Consortium for Political and Social Research (ICPSR)[56]	Various study designs	International	Metadata only	Study properties Text in study description Text in variable label
EU Joint Programme—Neurodegenerative Disease Research Global Cohort Portal (JPND)[57]	Cohorts	International	None	Study properties Text in study description Categories of information collected
Maternal, Infant, Child & Youth Research Network (MICYRN)[15]	Various study designs	Canada	None	Text in study description Categories of information collected
Medical Research Council Research Data Gateway[58]	Cohorts	United Kingdom	None	Study properties Text in study description Categories of information collected
National Archive of Computerized Data on Aging (NACDA)[59]	Various study designs	International	Metadata and data	Study properties Text in study description Text in variable label Categories of information collected
ONTOFORCE[60]	Various databases	International	None	Study properties Text in study description Categories of information collected
Portail Épidémiologie France[61]	Various study designs	France	None	Study properties Text in study description Categories of information collected
RAND Survey Metadata Repository[62]	Various study designs	International	None	-
Registry of Research Data Repositories (re3data.org)[63]	Various databases	International	None	Study properties Text in study description
Swedish National Data Service (SND)[64]	Various databases	Sweden	None	Study properties Text in study description
UK Data service[65]	Various study designs	United Kingdom	Metadata and data	Study properties Text in study description Text in variable label

### Maelstrom Research cataloguing toolkit

The Maelstrom Research cataloguing toolkit was built upon two main components: a metadata model and a suite of open-source software applications. Used together these components enable the creation of web-based searchable and customizable study and variable catalogues.

**Fig 1:**
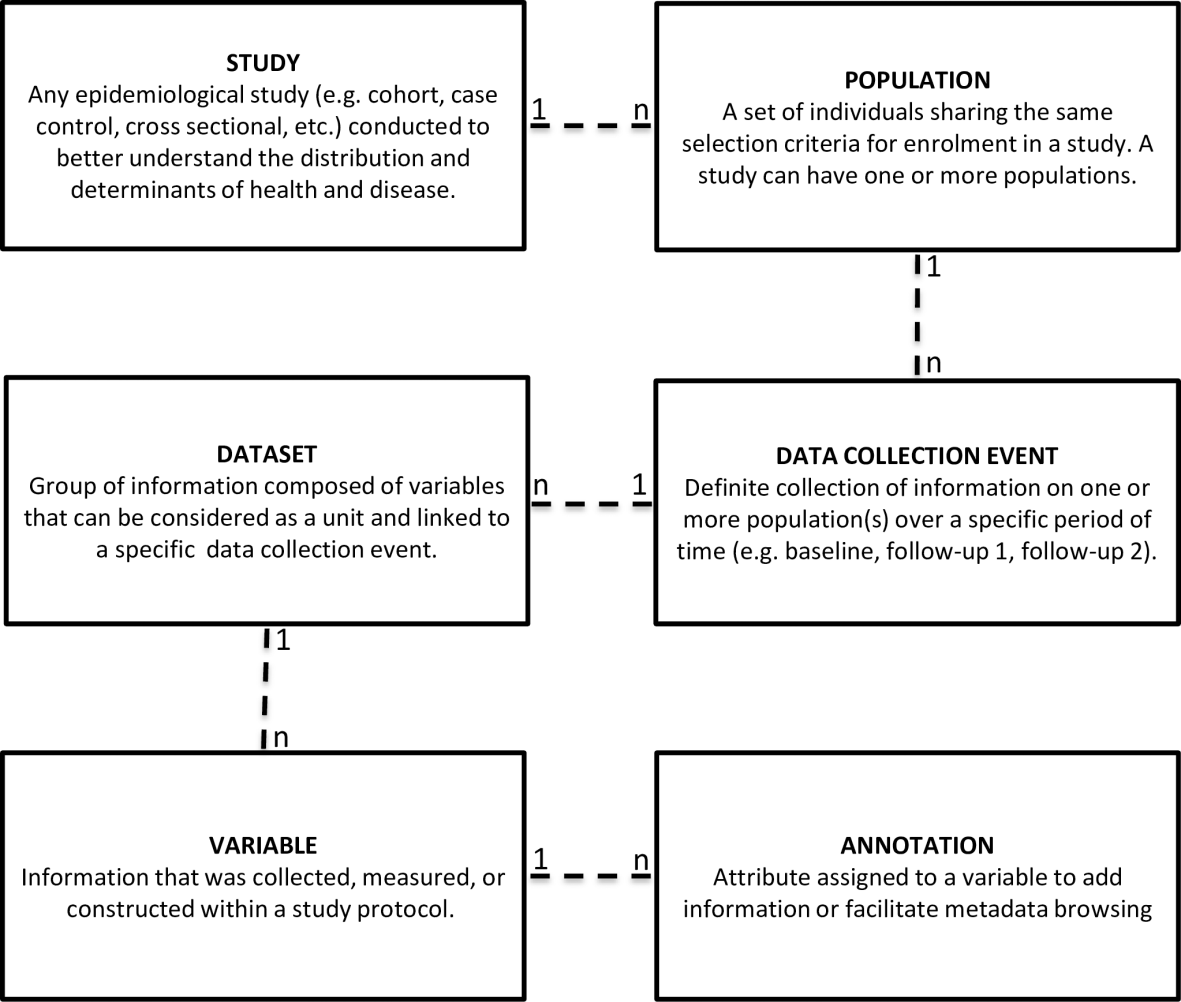
Conceptual model used to document cohort designs and variable content.

Fig 1 presents the conceptual model underlying the study-specific metadata fields. The model sets out specific fields to document: study outline; profiles of the subpopulations of participants; timing of data collection events (or participant follow-ups); and datasets/variables collected at each data collection event. It also includes the possibility to annotate variables with different classification schemes. Detailed information on the model and fields is provided in supporting information (S1 File).

The study outline includes the name, logo and website of the study, the list of investigators and contact persons, the objectives, timeline, and number of participants recruited and participants providing biological samples. It also provides information on access to data and samples. For each subpopulation of participants, information related to the recruitment of participants and selection criteria is included. Finally, documentation of each data collection event includes a general description, start and end dates, data sources and type of information collected.

Lists of variables collected at each data collection event can also be added. The dataset metadata fields include the name and a brief description of the dataset content. The variable metadata fields include the variable name and label, and if applicable, the code and label of each variable category. Additional variable-level metadata fields can also be documented, such as the specific question used to collect the data, or measurement units. Finally, variables can be annotated using various classification schemes. One such classification has been developed by our team to specifically serve the needs of toolkit users. The Maelstrom Research classification essentially allows categorizing all information collected by a study and is composed of 18 domains and 135 subdomains: Socio-demographic and economic characteristics (14 subdomains); Lifestyle and behaviours (14 subdomains); Birth, pregnancy and reproductive health history (5 subdomains); Perception of health, quality of life, development and functional limitations (6 subdomains); Diseases (20 subdomains; ICD-10); Symptoms and signs (9 subdomains; ICD-10); Medication and supplements (3 subdomains); Non-pharmacological interventions (7 subdomains); Health and community care services utilization (4 subdomains); Death (3 subdomains); Physical measures and assessments (11 subdomains); Laboratory measures (9 subdomains); Cognition, personality and psychological measures and assessments (4 subdomains); Life events, life plans, beliefs and values (4 subdomains); Preschool, school and work life (4 subdomains); Social environment and relationships (5 subdomains); Physical environment (7 subdomains); Administrative information (6 subdomains). A complete list of the subdomains is provided in supporting information (S1 File). This classification aims to facilitate browsing and extraction of variables by topics of interest and enables the generation of tables comparing domain-specific data collected across studies, subpopulations and data collection events (Table 3).

**Table 3:** Example of an interactive table comparing variables collected across subpopulations and data collection events of two birth cohorts for selected domains and subdomains of interest.

Study	Socio-demographic and economic characteristics		Lifestyle and behaviours		Diseases (ICD-10)
	Education (Number of variables)	Income, possessions, and benefits (Number of variables)	Tobacco (Number of variables)	Alcohol (Number of variables)	Pregnancy, childbirth and the puerperium (O00-O9A) (Number of variables)
**All Our Babies and All Our Families (AOB/F)**[66]					
*Mothers*					
24 weeks gestation	4	8	5	14	0
36 weeks gestation	0	3	7	9	0
4 months postpartum	0	6	5	4	0
1 year postpartum	0	8	5	4	0
3 years postpartum	1	2	5	4	0
*Children*					
1 year postpartum	0	2	0	0	0
**Alberta Pregnancy Outcomes and Nutrition (APrON)**[67]					
*Mothers*					
First trimester	1	1	15	15	49
Second trimester	0	0	7	5	35
Third trimester	0	7	6	6	29
12 weeks postpartum	0	0	6	6	34
24 weeks postpartum	0	0	2	2	0
12 months postpartum	0	0	3	3	0
*Partners*					
Second trimester	1	0	4	3	0
12 weeks postpartum	0	0	3	2	0
*Children*					
12 weeks postpartum	0	0	0	0	1

Two interoperable open source software applications were developed to provide study managers with easy-to-use tools to implement the conceptual model described above and create fully operational web-based metadata platforms[68]. First, Opal[69] is a software application used to store and manage both variable metadata (i.e. data dictionaries and codebooks) and individual participant data. Opal, used conjointly with ‘R’[70], allows users to import, validate, derive, analyze and export data and metadata. It allows upload of various data formats including CSV, SPSS, and SAS and can store data and metadata on an unlimited number of variables, which can be uniformly annotated using controlled lists of terms such as the variable classification described above. Secondly, the Mica[69] application makes use of this metadata to create web-based catalogues of one or more studies. Features include a user-friendly set of tools to manage and publish information on studies as prescribed by the Maelstrom Research conceptual model and metadata fields. Mica also supports management of demands for access to data. Opal and Mica software architecture and detailed functionalities have been described elsewhere [69].

Once metadata is published on a Mica-powered web portal, a powerful search engine allows users to identify studies and variables of interest and explore the potential to harmonize and co-analyze data across datasets. The search interface allows identifying studies based on study properties described in the metadata fields (e.g. number of participants, age range of the participants). It also enables identification of specific variables of interest by searching variable properties and text mining variable labels. Finally, domains and subdomains of the classification, or additional variables annotations (e.g. annotation of the measures or scales collected) can be used to extract variables of interest and generate comparison tables facilitating exploration of the harmonization potential across cohorts, subpopulations and data collection events (Table 3). All search results lead to specific entity pages describing the study network (where relevant), cohort, dataset and/or variable.

### Use case: The Maelstrom Research catalogue

In collaboration with partner networks, the Maelstrom Research team deployed the metadata cataloguing toolkit to create the Maelstrom Research catalogue (www.maelstrom-research.org)[71]. The catalogue currently includes 14 international networks, comprising more than 180 studies (mostly cohorts) from across the world, totalling more than 6,240,000 participants. Full data dictionaries are available for 102 of these studies, representing a total of over 760,000 annotated variables. New content is regularly added to the catalogue, increasing the number of studies and variables that can be searched within and across networks.

To ensure quality and standardization of the metadata documented across networks, standard operating procedures were implemented. Using information found in peer-reviewed journals or on institutional websites, the study outline is documented using Mica and validated by study investigators. Where possible, data dictionaries or codebooks are obtained, completed for missing information (e.g. missing labels) and formatted to be uploaded in Opal. Variables are then manually classified by domains and subdomains and validated with the help of an in-house automated classifier based on a machine learning method. When completed, study and variable-specific metadata are made publicly available on the Maelstrom Research website. For more information about the Maelstrom cataloguing procedures and rules, please refer to the supporting information (S2 File).

## Discussion

The Maelstrom Research cataloguing toolkit already serves the metadata dissemination needs of a number of international initiatives (Table 1). It distinguishes itself from network-specific catalogues and software solutions currently offered to the scientific community. Firstly, it is developed as an open source and generic tool to be used by a broad range of initiatives. Researchers can download the software to develop their own catalogue and make use (or not) of the metadata fields and variable classification proposed. Secondly, the suite of software applications can also be used in conjunction with ‘R’[70] to clean, manage, process, harmonize and analyze data. Therefore, the suite of software can also be used as a *global* solution for cohorts, allowing them to store and manage data as well as disseminate it to the scientific community. Thirdly, the tools offer the possibility to search studies and variables properties and annotations using many criteria and generate a broad range of search outputs. As the software is open source, these features can be customized to answer the needs of a given network. Finally, the toolkit was developed to serve the needs of study consortia and includes user-friendly features to easily estimate harmonization potential across studies, subpopulations and data collection events and document harmonized datasets generated across studies. The approach and software functionalities facilitating data harmonization and co-analysis have been previously published[69,72].

Even when using highly-performing tools, development of study and variable catalogues is challenging. The quality of a catalogue directly depends on the quality and comprehensiveness of the study-specific information documented. But, maintaining and providing access to understandable and comprehensive documentation to external users can be challenging for cohort investigators, and require resources not always available, particularly for the very small or long-established studies. In addition, the technical work required to build and maintain a catalogue is particularly demanding. For example, gathering comprehensive–and comparable—information on study designs necessitates the implementation of rigorous procedures and working in close collaboration with study investigators. Manual classification of variables is also a long and a tedious process prone to human error. Moreover, the information collected needs to be regularly revised to update metadata with new data collections. These challenges, among others, can lead to the creation of catalogues with partial or disparate information across studies, documenting limited subsets of variables (e.g. only information collected at baseline) or including only studies with data dictionaries available in a specific language or format. However, to truly optimize usage of available data and leverage scientific discovery, implementation of high quality metadata catalogues is essential. It is thus important to establish rigorous standard operating procedures when developing a catalogue, obtain sufficient financial support to implement and maintain it overtime, and where possible, ensure compatibility with other existing catalogues.

The toolkit developed by Maelstrom Research is certainly a useful resource, but it will need to keep evolving to properly respond to the increasing demand generated by its users. Incremental versions of the toolkit are regularly generated. However, it is essential to extend our community of developers and improve compatibility with complementary resources such as software aimed at assessing data quality, or efficient text mining resources supporting automated exploration of the harmonization potential across datasets.

We hope more initiatives will make use of the toolkit and allow this unique tool to achieve its full potential. In addition, through the Maelstrom Research catalogue we hope to offer the scientific community a central repository to document networks and member studies, and thus facilitate search for information across observational cohort studies worldwide.

## Supporting information

S1 File Study- and variable-specific metadata fields. (DOCX)

S2 File Procedures used to catalogue studies on the Maelstrom Research website. (DOCX)
