# Supplementary material for: Fostering population-based cohort data discovery: The Maelstrom Research cataloguing toolkit
Source: PLoS One. 2018 Jul 24;13(7):e0200926. doi: 10.1371/journal.pone.0200926 (PMC6057635; doi:10.1371/journal.pone.0200926)
Supplement: S1 File — (DOCX) [file pone.0200926.s001.docx]

**STUDY- AND VARIABLE-SPECIFIC METADATA FIELDS**

This section presents a description of the study-specific metadata model and fields proposed by Maelstrom Research. Studies or networks can apply the model as is or customize the metadata fields to fit their specific needs.

The study outline includes information on the general study characteristics, targeted populations and data collection events. Each study can be composed of one or more populations, and each population can be linked to one or more data collection events. Subsequently, one or more datasets containing multiple variables can be attached to each data collection event. Each variable can then be annotated using different classifications.

| **STUDY** | |
| --- | --- |
| **Field** | **Definition** |
| **Name** | Official name of the study. |
| **Acronym** | Study acronym. |
| **Website** | Study website URL. |
| **Investigators** | Name, affiliated institution and contact information of the principal investigators. |
| **Contacts** | Name, affiliated institution and contact information of the person to be contacted to have more information about the study. |
| **Objectives** | Main objectives of the study. |
| **Study timeline** | Date when first participants were recruited and study end date if the study is completed. |
| **Study design** | Information on specific study design.   1. Cohort 2. Case-control 3. Case only 4. Cross-sectional 5. Clinical trial 6. Other |
| **General information on follow-up** | Profile and frequency of participants’ follow-up  (*e.g. Participants are followed-up every 5 years*). |
| **Supplementary information about study design** | Additional information about study design  (*e.g. Subgroups of the population were intentionally over-sampled*). |
| **Recruitment target** | Type of participant units targeted by the study.   1. Individuals 2. Families 3. Other |
| **Number of participants** | Number of participants planned to be recruited. If the study is completed, the final number of participants. |
| **Number of participants with biological samples** | If the study is collecting biological samples, number of participants that should provide samples. If the study is completed, the final number of participants that provided biological samples. |
| **Supplementary information about number of participants** | Additional information about target number of participants  (*e.g. Additional biological samples will be collected for population 2*). |
| **Access** | Whether access to study data, biological samples or other study material by external researchers or third parties is allowed or foreseen. |
| **Marker paper(s)** | Bibliographic citation(s) which should be used to refer to the study and, if applicable, the paper’s Pubmed ID. |
| **Logo** | Logo used by the study. |
| **Documents** | Relevant documents about the study  (*e.g. Questionnaires, standard operating procedures, codebooks*). |

| **POPULATION** | |
| --- | --- |
| **Field** | **Definition** |
| **Name** | Name of the study population. |
| **Description** | A brief description of the population. |
| **Sources of recruitment** | Specification of the sources of recruitment.   1. General population (*volunteer enrolment, selected sample, random digit dialing*) 2. Specific population (*clinic patients, members of specific association, other specific population*) 3. Participants from existing studies 4. Other source |
| **Supplementary information about sources of recruitment** | Additional information about recruitment procedures  (*e.g. Participants were identified from the electoral register and general practice lists*). |
| **Selection criteria** | If relevant, specification for the following selection criteria of the participants.   1. Gender (*women or men*) 2. Age (*minimum age and maximum age*) 3. Residence (*country, territory or city*) 4. Pregnant women (*first trimester, second trimester, third trimester)* 5. Newborns 6. Twins 7. Ethnic origin 8. Health status 9. Other |
| **Supplementary information about selection criteria** | Additional information about selection criteria of the population  (e.g. *All subjects identified at baseline as affected by cognitive impairment without dementia were eligible for the longitudinal phase conducted after one year*). |
| **Number of participants** | Number of participants planned to be recruited for the population. If the study is completed, the final number of participants. |
| **Number of participants with biological samples** | If the study is collecting biological samples, number of participants that should provide samples for the population. If the study is completed, the final number of participants that provided biological samples. |
| **Supplementary information about number of participants** | Additional information about number of participants. Usually the number of participants for each wave of the study  (*e.g. Number of participants for each data collection event*  *Wave 1: 7175 participants*  *Wave 2: 3145 participants*  *Wave 3: 1733 participants*). |

| **DATA COLLECTION EVENT** | |
| --- | --- |
| **Field** | **Definition** |
| **Name** | Name of the data collection event. |
| **Description** | A brief description of the data collection event. |
| **Data collection event date** | Data collection start date and end date. |
| **Data sources** | Data sources from which the information is obtained.   1. Questionnaires 2. Physical measures 3. Cognitive measures 4. Biological samples (*blood, cord blood, buccal cells, tissues, saliva, urine, hair, nail, other*) 5. Administrative databases (*health databases, vital statistics databases, socioeconomic databases, environmental databases*) 6. Others (*e.g. medical files*) |

| **DATASET** | |
| --- | --- |
| **Field** | **Definition** |
| **Name** | Name of the dataset. |
| **Acronym** | Dataset acronym. |
| **Description** | Short description of the dataset specifying its content. |
| **Entity type** | What the data are about (usually the participant). |

| **VARIABLE** | |
| --- | --- |
| **Field** | **Definition** |
| **Dataset** | Name of the dataset in which the variable resides. |
| **Name** | Name of the variable. |
| **Label** | Short description of the variable specifying its content  (e.g. *Type of diabetes*).  Further information can be added in the **description** field. |
| **Description** | Additional information about the variable such as:   1. For variables collected by questionnaire, the question itself or any relevant information about the variable (e.g. *Have you ever been told by a doctor that you had diabetes?)* 2. For variables about physical/laboratory measures, any relevant information describing the context of measurement (e.g. *self-reported measure, measure by a trained professional*) or related to the protocol (e.g. *measure taken when the participant is at rest*) 3. For derived or constructed variables, any relevant information about the derivation or construction of the variable (e.g. *MMSE total score, total energy in Kcal per day derived from diet questionnaire*). |
| **Value type** | Type of variable:   1. Boolean (*two possible values (usually denoted true or false)*) 2. Date (*values written in a defined date format*) 3. Datetime (*values written in a defined date and time format*) 4. Decimal (*numerical values with a fractional component*) 5. Integer (*numerical values without a fractional component*) 6. Text (*alphanumerical values*) 7. Other types (*Point, line string, or polygon, etc.*)   (e.g. Type of diabetes has an integer value type: *1, 2, 3, 8, 9*). |
|  | **For continuous variables (where relevant)** |
| **Unit** | Measurement unit of the variable (e.g. *cm, mmol/L*). |
|  | **For categorical variables** |
| **Category name** | Value assigned to each variable category  (e.g. Type of diabetes has 5 categories: *1, 2, 3, 8, 9*). |
| **Category label** | Short description of the category  (e.g.:  1: *Type 1 diabetes*  2: *Type 2 diabetes*  3: *Gestational diabetes*  8: *Prefers not to answer*  9: *Missing*) |

| **VARIABLE ANNOTATION** |
| --- |
| The Maelstrom model supports usage of multiple variables annotations that can be used to better inform variable metadata content (e.g. name of the measure or standardized questionnaire used, source of the data, etc.). However, a classification index, was develop as complementary to the cataloguing toolkit. The Maelstrom Research classification can be used to facilitate variable search and was specifically developed to serve the needs of the platform users. It aims to facilitate selection of variables by topics of interest and generation of tables comparing variables content across studies, subpopulations and data collection events. This classification can theoretically be used to categorize all type of information collected by a study and is divided into 18 domains and 135 subdomains (see section below). Development of the classification was done through a series of workshops with cohorts’ investigators, computer scientists, statisticians and data managers. When is was possible, we used existing classifications, but is was not always the case. Some of the domains are thus based on international classification systems (e.g. International Classification of Diseases (ICD)) or are elements of existing classifications (International Classification of Functioning, Disability and Health (ICF)). However, for other domains no existing classification were available or could be used to classify variables provided by our partners. It was thus required to create new classes. |

| **MAELSTROM CLASSIFICATION: Domains and subdomains** |
| --- |
| **Socio-demographic and economic characteristics**  Age/birth date; Sex/gender; Twin; Marital/partner status; Family and household structure; Education; Residence; Birthplace; Citizenship and immigrant status; Ethnicity, race and religion; Language; Labour force and retirement; Income, possessions, and benefits; Other socio-demographic and economic characteristics |
| **Lifestyle and behaviours**  Tobacco; Alcohol; Drugs; Nutrition; Breastfeeding; Physical activity; Transportation; Personal hygiene; Sleep; Sexual behaviours and orientation; Leisure activities; Misbehaviour and criminality; Technological devices; Other and unspecified lifestyle information  **Birth, pregnancy and reproductive health history**  Puberty, menstruation, menopause and andropause; Contraception; Pregnancy, delivery, and birth; Fertility and sexual health; Other reproductive health-related information |
| **Perception of health, quality of life, development and functional limitations**  Perception of health; Quality of life; Life course development; Functional limitations; Use of assistive devices; Other perception of health, quality of life and functional limitation-related information |
| **Diseases (ICD-10)**  Certain infectious and parasitic diseases (A00-B99); Neoplasms (C00-D48); Diseases of the blood and blood-forming organs and certain disorders involving the immune mechanism (D50-D89); Endocrine, nutritional and metabolic diseases (E00-E90); Mental and behavioural disorders (F00-F99); Diseases of the nervous system (G00-G99); Diseases of the eye and adnexa (H00-H59); Diseases of the ear and mastoid process (H60-H95); Diseases of the circulatory system (I00-I99); Diseases of the respiratory system (J00-J99); Diseases of the digestive system (K00-K93); Diseases of the skin and subcutaneous tissue (L00-L99); Diseases of the musculoskeletal system and connective tissue (M00-M99); Diseases of the genitourinary system (N00-N99); Pregnancy, childbirth and the puerperium (O00-O9A); Certain conditions originating in the perinatal period (P00-P96); Congenital malformations, deformations and chromosomal abnormalities (Q00-Q99); Injury, poisoning and certain other consequences of external causes (S00-T98); External causes of morbidity and mortality (V01-Y98); Diseases without precise specification or falling into multiple categories |
| **Symptoms and signs (ICD-10)**  Symptoms and signs involving the circulatory and respiratory systems (R00-R09); Symptoms and signs involving the digestive system and abdomen (R10-R19); Symptoms and signs involving the skin and subcutaneous tissue (R20-R23); Symptoms and signs involving nervous and musculoskeletal systems (R25-R29); Symptoms and signs involving the urinary system (R30-R39); Symptoms and signs involving cognition, perception, emotional state and behaviour (R40-R46); Symptoms and signs involving speech and voice (R47-R49); General symptoms and signs (R50-R69); Symptoms related to multiple categories |
| **Medication and supplements**  Medication and supplement intake; Posology and protocol of administration; Other and unspecified pharmacological interventions |
| **Non-pharmacological interventions**  Surgical interventions; Radiological interventions; Physical therapy interventions; Cognitive, psychological and sensory interventions; Educational and health promotion interventions; Laboratory diagnosis interventions; Other and unspecified non-pharmacological interventions |
| **Health and community care services utilization**  Visits to health professionals; Hospitalizations; Community and social care; Other health and community care  **Death**  Vital status; Cause of death; Other end of life or death-related information |
|  |
|  |
|  |
| **Physical measures and assessments**  Physical characteristics; Anthropometry; Circulation and respiration; Muscles, skeleton and mobility; Sensory and pain; Brain and nerves; Skin and subcutaneous tissue; Speech and voice; Digestion; Reproduction; Other physical measures and assessments  **Laboratory measures**  Hematology; Biochemistry; Microbiology; Virology; Immunology; Toxicology; Histology; Genomics; Other laboratory measures |
| **Cognition, personality and psychological measures and assessments**  Cognitive functioning; Personality; Psychological distress and emotions; Other psychological measures and assessments |
|  |
| **Life events, life plans, beliefs and values**  Life events; Life plans; Beliefs and values; Other life events, plans and beliefs  **Preschool, school and work life**  Preschool life; School life; Work life; Other preschool, school or work life-related information |
| **Social environment and relationships**  Social network; Social participation; Social support; Parenting and familial environment; Other social environment characteristics |
| **Physical environment**  Housing characteristics; Built environment/neighbourhood characteristics; Workplace characteristics; Radiation exposure; Chemical exposure; Biological exposure; Other physical environment characteristics |
| **Administrative information**  Identifiers; Date and time-related information; Questionnaire and interview-related information; Physical and cognitive measures and biosample-related information; Data and sample collection center-related information; Other administrative information |
