# Supplementary material for: Fostering population-based cohort data discovery: The Maelstrom Research cataloguing toolkit
Source: PLoS One. 2018 Jul 24;13(7):e0200926. doi: 10.1371/journal.pone.0200926 (PMC6057635; doi:10.1371/journal.pone.0200926)
Supplement: S2 File — (DOCX) [file pone.0200926.s002.docx]

**PROCEDURES USED TO CATALOGUE STUDIES ON THE MAELSTROM RESEARCH WEBSITE**

The cataloguing process used by the Maelstrom Research team to document an individual study on its catalogue ([www.maelstrom-research.org](http://www.maelstrom-research.org)) is divided in 3 steps: study description, variables documentation and variables annotation.

**Step 1: Completion of the study description**

Aim: Document the study design, targeted population(s) and data collection event(s).

Procedures:

- Gather information about the study from different sources including published papers and study website.
- Complete the fields of the study description model available in Mica.
- Ensure validation of the study description by a second person to ascertain the adequacy and quality of its content.
- Obtain validation and, if required, additional information from the study investigators.
- Make any required modifications and publish the study description on the Maelstrom Research website.

**Step 2: Documentation of the study variables**

Aim: Generate standardized variable dictionaries.

Procedures:

- Obtain the questionnaires and data dictionary from the study investigator. The data dictionary can be in different formats (SPSS, Excel, csv, etc.).
- Format the data dictionary to be compatible with Opal.
- Evaluate completeness of the data dictionary content.
- Correct any missing or unclear information with the help of the questionnaires (label, category codes and labels). Variables should at least have a name, a label, and if applicable, codes and labels for categories. If impossible, ask study investigators to add the missing information and send back the complete data dictionary.

**Step 3: Annotation of variables by domains and sub-domains**

Aim: Classify each study variable in at least one domain and subdomain of the Maelstrom Research classification.

Procedures:

- First research assistant: attribution of each variable to one or more subdomains of the areas of information with the help of the questionnaires and the information documented in the previous cataloguing steps. The context surrounding the variable should prevail on blindly applying the rules.
- Validation of the classification using an in-house automated classifier based on a machine learning method. This, to identify discrepancies between the human and the machine annotations.
- Second research assistant: validation of all variables for which a divergence was observed and where relevant, suggestion of modifications to the initial classification.
- First research assistant: review of the suggested modifications.
- If disagreement on the classification of a variable remains, group discussion to take final decision.
- Upload annotated variables on Opal and publish variables data dictionaries and related annotation on the Maelstrom Research website.
